# You can keep your coat on

**DOI:** 10.7554/eLife.69887

**Published:** 2021-06-01

**Authors:** Gregory J Bedwell, Alan N Engelman

**Affiliations:** 1 Department of Cancer Immunology and Virology, Dana-Farber Cancer InstituteBostonUnited States; 2 Department of Medicine, Harvard Medical SchoolBostonUnited States

**Keywords:** HIV-1, uncoating, reverse transcription, DNA labeling, live cell imaging, CLEM, Human, Virus

## Abstract

High-resolution imaging techniques reveal new insights into the actions of the retrovirus HIV-1 inside host cells.

**Related research article** Müller TG, Zila V, Peters K, Schifferdecker S, Stanic M, Lucic B, Laketa V, Lusic M, Müller B, Kräusslich HG. 2021. HIV-1 uncoating by release of viral cDNA from capsid-like structures in the nucleus of infected cells. *eLife* **10**:e64776. doi: 10.7554/eLife.64776

Viruses are pathogens that rely on hosts to produce multiple copies of themselves. Retroviruses, such as HIV-1, have a unique way of integrating their genes into the DNA of a host cell. Since their genetic information is stored as RNA, a retrovirus must first undergo a process called reverse transcription. This process, which is catalyzed by an enzyme called reverse transcriptase, is followed by a second step called integration (catalyzed by integrase), which results in the viral DNA being inserted into the genome of the host cell. Although the mechanical functions of these two enzymes are well understood, relatively little is known about the transition between reverse transcription and integration during HIV-1 infection.

Much of the research into these processes has centered on the capsid protein, which self-associates to form a honeycomb-like container that surrounds the genetic material inside the retrovirus (Bedwell and Engelman, 2021). Once the retrovirus has entered a cell, the capsid container protects the RNA and the two enzymes from the antiviral molecular machinery in the cell cytoplasm, allowing reverse transcription to proceed without interruption (Yamashita and Engelman, 2017). At the same time, various cellular proteins bind to the outside of the capsid in order to facilitate it being trafficked to the nucleus, where the DNA of the host cell resides.

Once reverse transcription has been completed, the capsid is degraded to release the viral DNA from its protective container. Until recently, it was assumed that this process – which is called uncoating – might be a prerequisite for the viral DNA to enter the nucleus because it was thought that the HIV-1 capsid was too large to pass through the nuclear pore complex (which is the doorway through which biomolecules pass to enter the nucleus; Yamashita and Engelman, 2017; Arhel et al., 2007; Francis and Melikyan, 2018). However, recent studies have found evidence for capsid lattices or intact capsid-like structures inside the nucleus (Burdick et al., 2020; Dharan et al., 2020; Selyutina et al., 2020), with one study even suggesting that the intact capsid is able to pass through the nuclear pore complex (Zila et al., 2021; Figure 1). Now, in eLife, Hans-Georg Kräusslich and colleagues at the University Hospital Heidelberg and the German Center for Infection Research – including Thorsten Müller as first author – report new details on the timing, localization and mechanism of HIV-1 uncoating (Müller et al., 2021).

**Figure 1.**
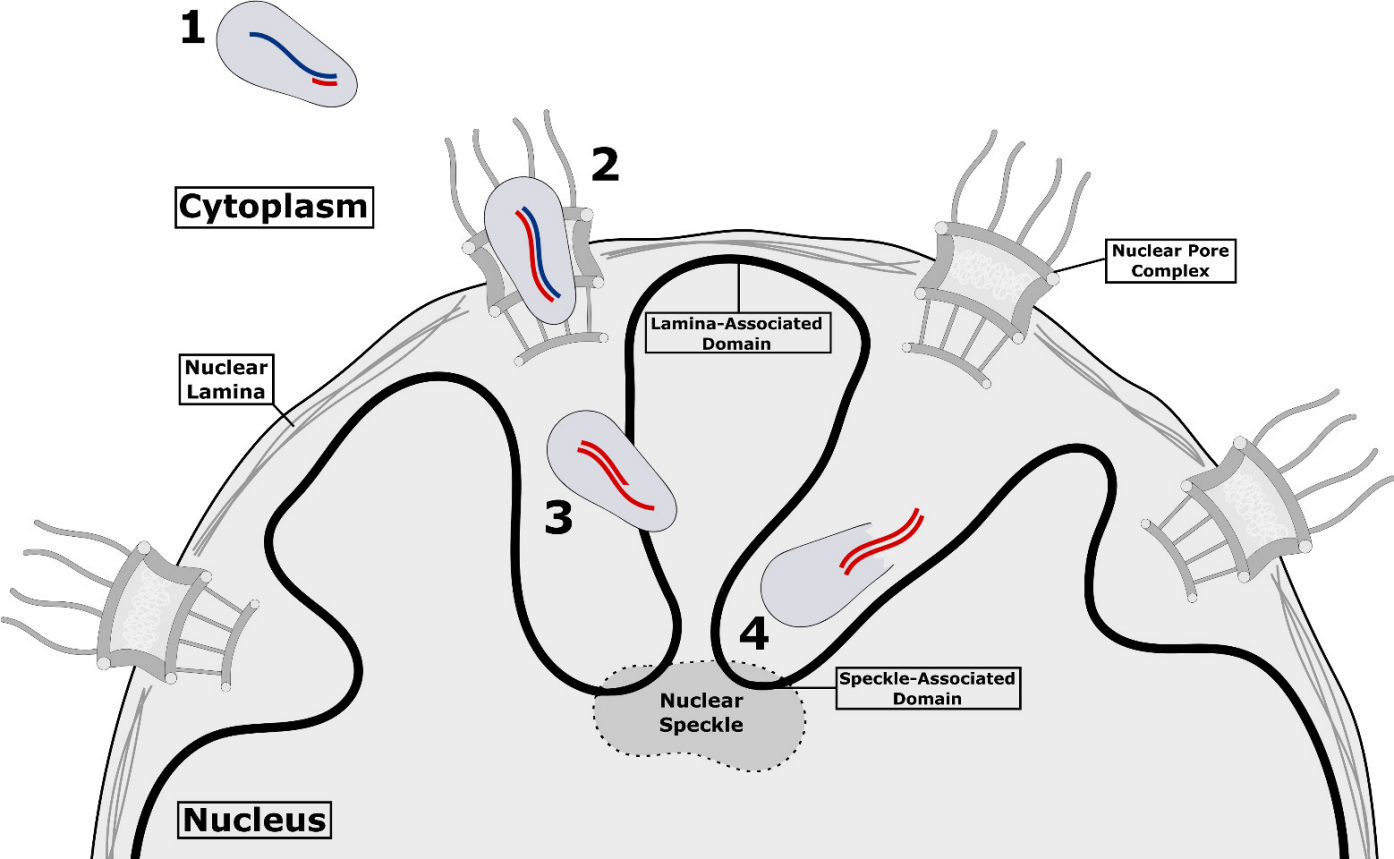
HIV-1 inside a host cell. After HIV-1 has reached the cytoplasm of the host cell (1), reverse transcription begins within the virus capsid, which is still intact: viral RNA in shown in blue, and the newly synthesized viral DNA is shown in red. Reverse transcription continues as the capsid passes through the nuclear pore complex (2) and into the nucleus (3). Once the process of reverse transcription has been completed, the forces exerted by the double-stranded DNA (double red lines) compromise the structural integrity of the capsid, creating a gap through which the DNA can escape (4) and go on to be integrated in the DNA of the host cell (not shown). The breakdown of the capsid and release of the DNA is referred to as uncoating. Integration occurs in chromatin (solid black line) near nuclear speckles (speckle-associated domains) and away from nuclear lamina (lamina-associated domains). It is currently unclear if there are similar preferred sites for uncoating.

Müller et al. used different fluorescent labels to identify distinct events during HIV-1 replication. The ANCHOR labeling system – which employs a fluorescently labeled bacterial protein to bind a specific DNA sequence engineered into the HIV-1 genome – enabled them to distinguish between the initial reverse-transcription products made comparatively early during infection, and the more complete, double-stranded DNA that is accessible only after uncoating has happened. Müller et al. also tagged the incoming virus with a fluorescently labeled integrase protein in order to study the kinetics of uncoating. These experiments revealed that complete viral DNA was mostly tagged after nuclear import, suggesting that the capsid remained intact as it entered the nucleus, and that uncoating took place thereafter. Moreover, uncoating began in the nucleus as early as eight hours after infection with HIV-1 (Figure 1).

These observations suggest little – if any – loss of the capsid protein prior to nuclear import. Consistent with this interpretation, high-resolution imaging techniques revealed intact capsid-like structures at sites close to ANCHOR-labeled viral DNA. Tomographic reconstructions of these structures provided a unique snapshot of the HIV-1 uncoating process, showing what appeared to be holes in capsid-like structures that did not contain viral DNA. This quite striking finding is consistent with a previous model of uncoating in which reverse transcription leads to the generation of mechanical forces that compromise the structural integrity of the capsid (Figure 1; Rankovic et al., 2017). In many ways, the capsid is like an egg from which the integration-competent viral DNA is hatched!

The work of Müller et al. fills in some of the gaps in our understanding of the early stages of HIV-1 replication. We now have a clearer picture of how and when the final integration-competent complex can be released from the capsid shell. An important next step will be to unravel the link between uncoating and integration.

In recent years, it has become clear that integration tends to take place at chromatin regions that are close to structures called nuclear speckles and away from the nuclear lamina (Figure 1; Bedwell and Engelman, 2021). It is possible, perhaps even likely, that uncoating also occurs at or near these same chromatin regions. Alternatively, a post-uncoating trafficking event might help guide the integration complex to these regions. A theory that can link reverse transcription, uncoating and integration will clarify the paths taken during HIV-1 infection which, in turn, could inform the development of new antiviral strategies.
